# Iron Effects on *Clostridioides difficile* Toxin Production and Antimicrobial Susceptibilities

**DOI:** 10.3390/antibiotics11050537

**Published:** 2022-04-19

**Authors:** Jason Yamaki, Swati Chawla, Shirley Tong, Kate Alison Lozada, Sun Yang

**Affiliations:** 1 Department of Pharmacy Practice, Chapman University School of Pharmacy, Irvine, CA 92618, USA; yamaki@chapman.edu (J.Y.); chawlaswati@hotmail.com (S.C.); 2 Department of Biomedical and Pharmaceutical Sciences, Chapman University School of Pharmacy, Irvine, CA 92618, USA; fong149@mail.chapman.edu (S.T.); klozada@chapman.edu (K.A.L.)

**Keywords:** *C. difficile* infection, iron overload, iron chelator, metronidazole, vancomycin, deferoxamine

## Abstract

Despite the benefits of red blood cell (RBC) transfusion therapy, it can render patients vulnerable to iron overload. The excess iron deposits in various body tissues cause severe complications and organ damage such as cardiotoxicity and mold infections. *Clostridioides difficile* infection (CDI) is the most common cause of nosocomial diarrhea among cancer patients and is associated with significant morbidity and mortality. Our study aims to determine the role of iron overload and the effects of iron chelators on CDI. Our results demonstrated that iron (Fe^3+^) stimulated the growth of *C. difficile* with increased colony formation units (CFU) in a dose-dependent manner. Exposure to excess iron also increased the gene expression levels of *tcdA* and *tcdB*. The production of *C. difficile* toxin A, necessary for the pathogenesis of *C. difficile*, was also elevated after iron treatment. In the presence of excess iron, *C. difficile* becomes less susceptible to metronidazole with significantly elevated minimum inhibitory concentration (MIC) but remains susceptible to vancomycin. Iron-stimulated colony formation and production of *C. difficile* toxins were effectively diminished by iron chelator deferoxamine co-treatment. Incorporating iron overload status as a potential factor in developing a risk prediction model of CDI and antibiotic treatment response may aid clinical practitioners in optimizing CDI management in oncology patients.

## 1. Introduction

*Clostridioides difficile*, a Gram-positive, spore-forming, obligately anaerobic bacterium, is the leading cause of healthcare-associated infectious diarrhea, ranging from mild diarrhea to pseudomembranous colitis and toxic megacolon, with an increasing incidence and associated mortality [1]. *C. difficile* infection (CDI) is a toxin-mediated disease, and the production of two clostridial toxins (TcdA and TcdB) are considered the cause of CDI symptoms by traditional *C. difficile* strains. In the early 2000s, a new epidemic strain BI/NAP1 ribotype 027 began circulating and causing severe infections [2]. This strain was characterized by being fluoroquinolone-resistant, having a third toxin known as binary toxin, and producing increased amounts of TcdA and TcdB due to deletion of the *tcdC* gene, which is a negative regulator of toxin production [3,4].

*C. difficile* can cause infections in any patient; however, there are specific risk factors that are strongly associated with CDI infection. These factors include exposure to antibiotic therapy or proton pump inhibitors, age 65 and over, ulcerative colitis, immunosuppression, and receipt of chemotherapy [5,6]. The last group of high-risk patients is particularly vulnerable since oncology patients are particularly at risk due to immunosuppression, broad-spectrum antibiotic use, chemotherapy, and frequent hospitalization, resulting in significant morbidity and mortality in this population [7]. Additionally, CDI has also been associated with an increased risk of death and prolonged hospitalization in children [8,9,10,11]. Recent studies have demonstrated that *C. difficile* strains utilize various exogenous organic and inorganic iron sources for growth, and mutations within the ferric uptake regulator gene have been reported to confer metronidazole (MTZ) resistance in *C. difficile* [12].

Iron is an essential micronutrient for microorganisms and humans. Iron is bound to protein in the human body, such as heme compounds (hemoglobin), heme enzymes, or nonheme compounds (ferritin). The body uses iron to form the proteins or enzymes essential for electron transfer and oxidation–reduction reactions, such as oxygen transport protein hemoglobin. Since iron is highly bound and conserved to the iron-binding protein ferritin, serum ferritin level is commonly used to estimate iron stores in the body [13]. Oncology patients have a high risk of iron overload resulting from frequent red blood cell transfusions [14]. Each infused RBC unit contains approximately 200–250 mg of iron [15]. Iron typically binds to transferrin, an iron storage protein. However, when transferrin is saturated, free iron circulates in the body and is deposited into tissues. Given the fact that humans have no physiologic mechanism for excreting excess iron, the unbound free iron catalyzes the formation of reactive oxygen species, which leads to organ damage [16,17].

Moreover, iron has been shown to stimulate the growth of many pathogenic microorganisms such as *Mycobacterium tuberculosis* [18,19]. Invading microbes including *Escherichia coli*, *Klebsiella pneumoniae*, *Staphylococcus aureus*, and *Salmonella* species have developed multiple strategies to utilize iron as a nutrient, resulting in pathogen proliferation and virulence [20,21,22,23,24,25]. Recent studies have also demonstrated a significant relationship between iron overload, infectious complications, and poor prognosis in oncology patients post-hematopoietic stem cell transplantation. The prevalence of severe infectious events such as bacteremia, invasive aspergillosis, and mucormycosis was significantly higher in patients that presented with iron overload [26,27].

In this study, we report that in vitro, excess iron stimulates the growth of *C. difficile* and increases the expression of toxin A and B mRNA (*tcdA* and *tcdB*). Exposure to excess iron also leads to metronidazole tolerance, as measured by increasing minimum inhibitory concentrations (MICs). The iron chelator, deferoxamine (DFO), returned the metronidazole MIC to baseline and effectively inhibited the growth and toxin production of *C. difficile* induced by iron.

## 2. Results

### 2.1. Iron Stimulates C. difficile Growth and Toxin Production In Vitro

**Figure 1:**
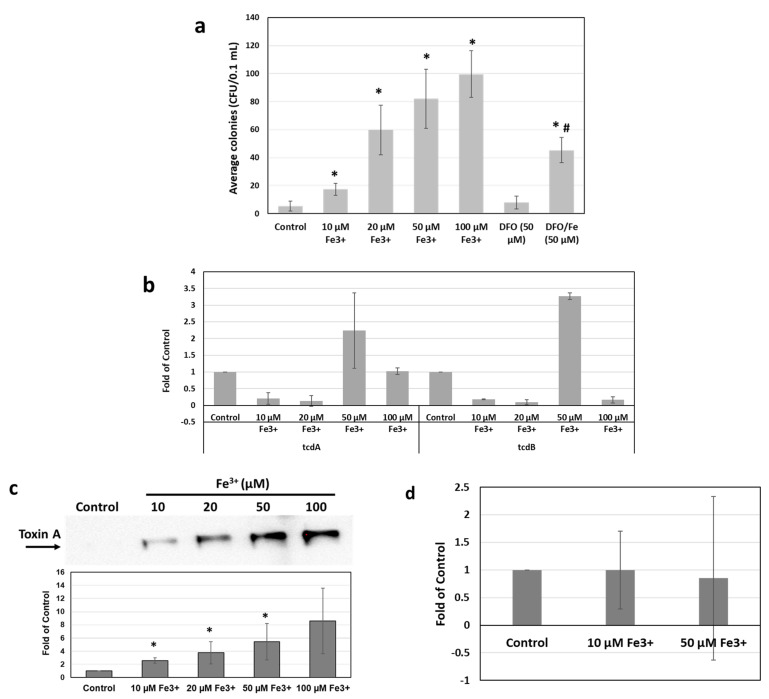
Effects of iron on *C. difficile* colony formation and production of toxins: (**a**) the increase in colony formation units following Fe^3+^ treatments was suppressed by iron chelator DFO co-treatment in *C. difficile* strain 9689. The results are presented as the average of colonies formed from three plates. *, *p* < 0.05 compared to vehicle control; #, *p* < 0.05 compared to 50 µM Fe^3+^; (**b**,**c**) *C. difficile* toxins expressions were increased by iron exposure. After 16 h treatments, total RNA or concentrated bacterial supernatants were collected for qRT–PCR (**b**) and Western Blot (**c**) analyses. The mRNA expression levels of *tcdA* and *tcdB* were normalized by GAPDH expression and standardized by vehicle control. After treatments, equal volumes of clear concentrated supernatants were subjected to SDS–PAGE Western Blotting to determine the toxin A levels. *, *p* < 0.05 compared to vehicle control; (**d**) the expression levels of toxin A after iron treatment detected by Western Blot were further adjusted by CFU values in the 9689 strain. The results are presented as the average of three experiments.

The effects of Fe^3+^ on bacterial growth were assessed by colony-forming units (CFUs). As shown in Figure 1a, a significant increase in CFU/mL after 16 h incubation was evident in the 9689 strain grown in media supplemented with FeCl_3_. The increase in CFU was observed in the presence of Fe^3+^ as low as 10 µM (3.5-fold of control) and peaked at 100 µM in a dose-dependent manner to 13.8-fold of control. Co-incubation with DFO significantly reduced the CFU in the presence of 50 µM Fe^3+^, compared with FeCl_3_ alone (*p* < 0.05). However, the average CFU with DFO and FeCl_3_ co-treatment remained higher than the control. The induction of CFU was also evident in the R20291 strain but to a lesser extent (~1.9-fold of control, Supplementary Figure S1).

The gene expression levels of *tcdA* and *tcdB* were also detected by qRT–PCR (Figure 1b) [28] after iron treatments for 16 h. As shown in Figure 1b, the *tcdA* and *tcdB* expression levels were significantly increased by 50 µM Fe^3+^ treatment in the 9689 strain, where mRNA levels of *tcdA* and *tcdB* increased to 2.2- and 3.3-fold of the non-treatment control, respectively (*p* < 0.05).

After culturing at various concentrations of Fe^3+^ for 16 h, the bacterial culture supernatants were collected and concentrated using a 3K concentration spin. Equal volumes of samples were subjected to Western Blot analysis, and the band densities were measured (Figure 1c). The presence of iron increased strain 9689 toxin A production in a dose-dependent manner, resulting in 2.6-, 3.8-, 5.5-, and 8.6-fold change with concentrations of 10 µM, 20 µM, 50 µM, and 100 µM of Fe^3+^, respectively. However, when normalizing the data to CFU, no significant change was observed after Fe^3+^ treatment, compared with the control (Figure 1d).

### 2.2. Effects of Iron Chelation on Metronidazole MIC Profiles of C. difficile Strains

MICs of MTZ in *C. difficile* strains were determined using microbroth dilution assay according to modified Clinical and Laboratory Standards Institute (CLSI) guidelines for testing anaerobes [29]. Two *C. difficile* strains with different virulence and clinical prevalence (Supplementary Table S1) were tested to determine the susceptibility of antibiotics in the absence or presence of excess Fe^3+^. The *C. difficile* strains tested included the historical 9689 and the clinical hypervirulent R20291 strains. The 9689 strain is susceptible to MTZ, while the R20291 strain is MTZ-non-susceptible. In our study, R20291 showed a marked decrease in the susceptibility to MTZ, and MICs were up to 64 µg/mL under the standard treatment conditions.

**Figure 2:**
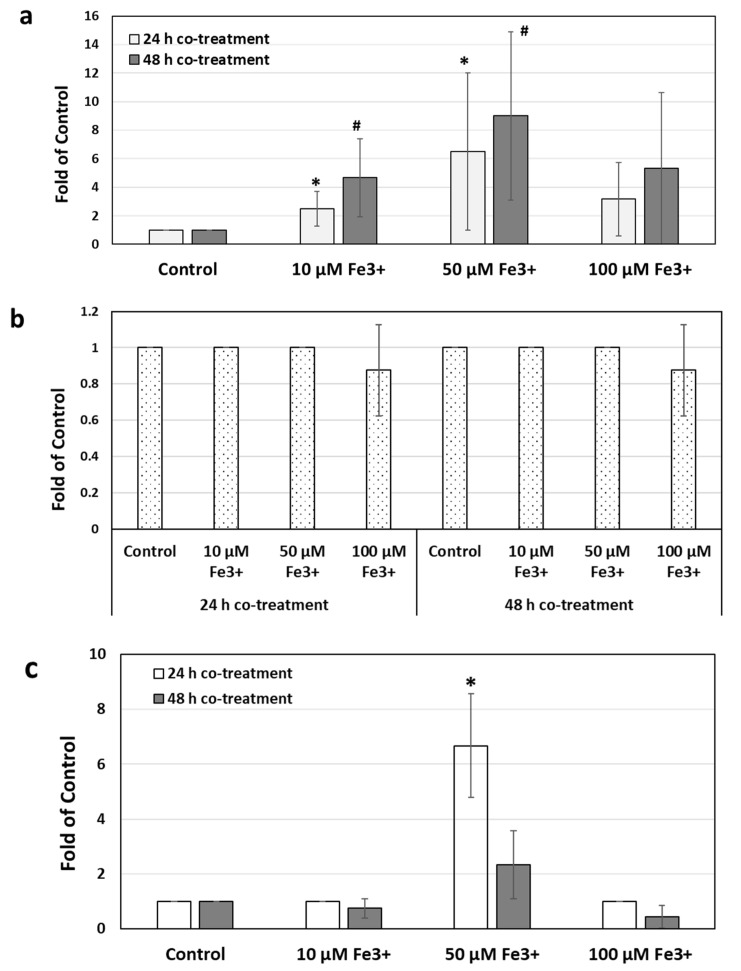
The presence of iron affected the MICs of metronidazole in different *C. difficile* strains. The effect of Fe^3+^ on metronidazole MICs (**a**), vancomycin MICs (**b**), and fidaxomicin MICs (**c**) in *C. difficile* strain 9689. The antibiotic susceptibility was determined by the minimum inhibitory concentration (MIC). Each experiment was repeated at least three times independently and presented as mean ± SD. *, *p* < 0.05 compared to 24 h control; #, *p* < 0.05 compared to 48 h control.

In the presence of excess iron, the MICs of MTZ were significantly increased in *C. difficile* strain 9689 (Figure 2a and Supplementary Table S2). After 48 h incubation with 50 µM Fe^3+^, the average MIC increased from 0.17 µg/mL to 1.38 µg/mL, and in some replicates, the MIC was up to 2 µg/mL, which is at the breakpoint indicated by the European Committee on Antimicrobial Susceptibility Testing (EUCAST) for MTZ and *C. difficile*. The MTZ MICs were highest after co-incubation with 50 µM Fe^3+^. Increasing iron concentration to 100 µM did not increase the MICs any further. In the MTZ-non-susceptible strain, R20291, no changes in susceptibility to metronidazole were observed for 24 h or 48 h under any of the treatment concentrations (Supplementary Table S2).

Fidaxomicin MICs also significantly increased in the presence of Fe^3+^. As shown in Figure 2c, after 24 h incubation, Fe^3+^ (50 µM) increased the MIC of fidaxomicin to 6.7-fold of control in the *C. difficile* 9689 strain but were still at values < 1 µg/mL, which would be considered susceptible. In strain R20291, there was an eightfold increase in MIC at 48 h regardless of the presence of iron and an approximately twofold increase under 10 µM and 50 µM iron concentrations. The fidaxomicin MIC was consistently 1 µg/mL at 24 h under control conditions and tested three different iron concentrations (Supplementary Table S4). However, at 48 h, the MICs of control increased to 8 µg/mL, and under 50 µM Fe^3+^, the MIC further increased to 16 µg/mL. Notably, a MIC value higher than 8 µg/mL would be considered to have reduced fidaxomicin susceptibility [30,31]. Interestingly, both the tested *C. difficile* strains remained susceptible to vancomycin (Figure 2b and Supplementary Table S3), and the MICs of vancomycin in the presence of excess iron were not significantly different, compared with the control ones without FeCl_3_ (*p* > 0.05) (Figure 2b). No changes in MICs were observed even with prolonged incubation for 48 h (Supplementary Table S4).

### 2.3. Effects of Iron Chelation on Iron-Induced Toxin Production and Metronidazole Resistance

**Figure 3:**
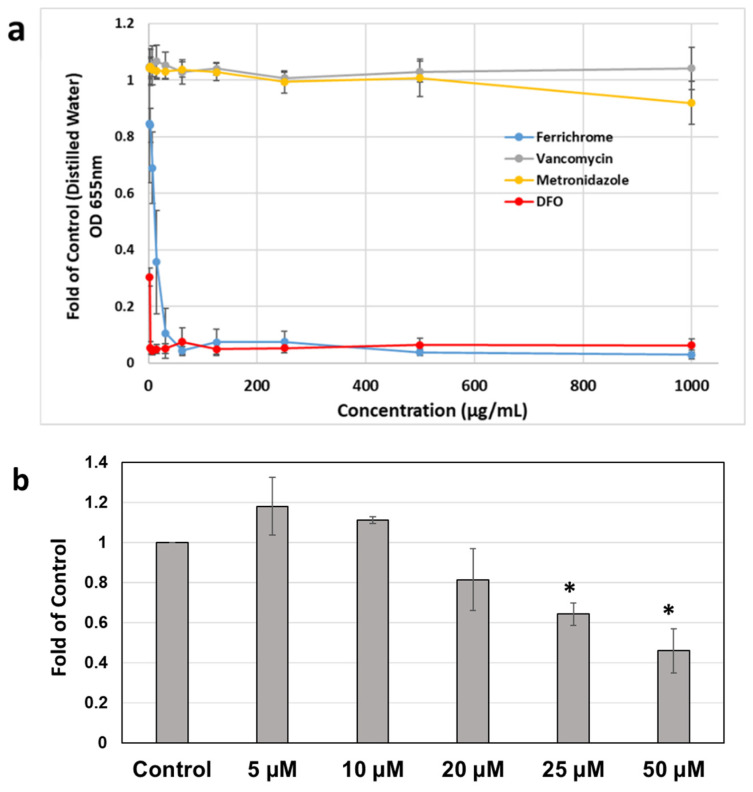

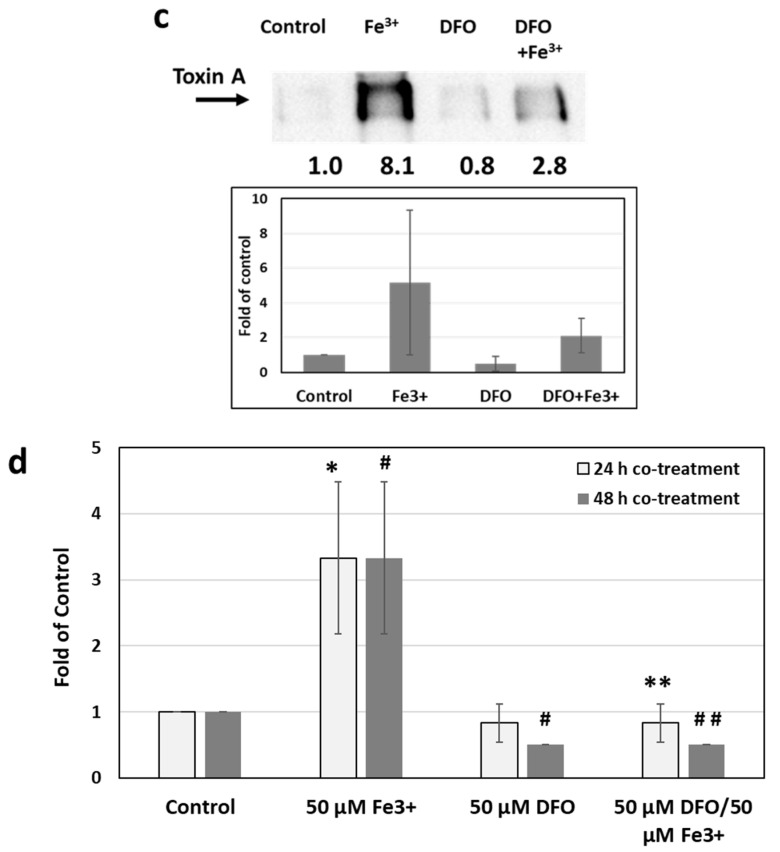
Effects of iron chelators on CDI: (**a**) the iron-chelating activity of deferoxamine (DFO) was determined using the CAS colorimetric assay as described before [32,33]. Reduction in A650 occurs when a strong chelator removes the iron from the chrome azurol sulfate dye. Ferrichrome was used as a positive control; (**b**) decreased colony formation of *C. difficile* strain 9689 after DFO treatments for 16 h. The results were presented as the average of colonies formed from three plates. *, *p* < 0.05 compared to vehicle control; (**c**) increased production of *C. difficile* toxin A after iron exposure was diminished by DFO co-treatment in strain 9689. Concentrated culture supernatant after treatments were subjected to Western Blot analysis. Experiments were repeated at least three times independently and presented as band density (mean ± SD); (**d**) effect of DFO on the susceptibility of *C. difficile* to metronidazole. The antibiotic susceptibility was determined by the MICs. Each experiment was repeated at least three times independently and presented as mean ± SD. *, *p* < 0.05 compared to 24 h control; #, *p* < 0.05 compared to 48 h control; **, *p* < 0.05 compared to 24 h 50 µM Fe^3+^; ##, *p* < 0.05 compared to 48 h 50 µM Fe^3+^.

As the above experiments demonstrated that the presence of iron could lead to increases in CFU/mL, toxin mRNA and production, and increases in MICs of MTZ, the effects of iron chelators were explored. The iron chelator DFO exhibited potent iron chelation as determined by chrome azurol S (CAS) colorimetric assay (Figure 3a) [32], where DFO formed complexes with iron resulting in a significant decrease in the optical density of the solution. The cyclic hexapeptide, ferrichrome, possessing a high ability to form iron complexes, was used as a positive control. Vancomycin exhibited no iron chelation activity at any of the tested concentrations, while metronidazole exhibited some iron chelation ability only evident at a high concentration of 1000 μg/mL.

Treatment of DFO alone effectively reduced colony formation in the *C. difficile* 9689 strain in a dose-dependent manner (Figure 3b). After exposure to DFO (50 µM) for 16 h, the CFU reduced to 45.8% of control (*p* < 0.05). Iron-induced toxin A production was also diminished by DFO, with the expression levels of toxin A reduced from 5.2-fold to 2.1-fold of control after DFO co-treatment (Figure 3c). Consistently, co-incubation with DFO sensitized *C. difficile* to MTZ treatment and effectively reduced the MICs of MTZ in the presence of excess iron (*p* < 0.05, Figure 3d).

Finally, to determine if removing free Fe^3+^ via chelation with DFO resulted in a reversion to baseline susceptibility, strain 9689 was co-treated with 50 µM DFO and MTZ for 24 h and 48 h. As shown in Figure 3d, the MIC had increased by 3.3-fold of control after exposure to 50 µM of FeCl_3_. DFO co-treatment effectively re-sensitized *C. difficile* to MTZ, and the MIC was reduced to control values after 24 h treatment and even lower than that of control after 48 h treatment.

## 3. Discussion

Despite the benefits of red blood cell (RBC) transfusion therapy, it renders patients vulnerable to iron overload [14]. Iron loading from blood transfusions can be estimated from the total number of units of red blood cells given, with each infused unit of RBCs containing approximately 200 to 250 mg of iron [15]. The National Comprehensive Cancer Network (NCCN) Clinical Practice Guideline recommends adjustment for each patient to maintain the lowest hemoglobin level sufficient to avoid RBC transfusion due to the risk of iron overload [34].

Iron is an essential co-factor in many reactions and leads to many transcriptional changes [35]. Under normal conditions, there is typically low iron availability within the body and serum, as iron is tightly regulated within the host. However, *C. difficile* and other bacteria produce siderophores that bind iron in the environment for subsequent uptake through ABC transporters [36]. In addition, Hastie et al. found that *C. difficile* growth is less robust and considerably decreased under iron-limited media than the standard media [37]. Consistently, our study demonstrated that the growth and toxin production of *C. difficile* were significantly stimulated by exposure to excess iron. Both observations further confirm iron’s nutritional role in microorganism growth, also described in other bacteria and fungi pathogenesis [38,39].

Until recently, the underlying mechanism of *C. difficile* resistance to metronidazole was not well defined. An earlier proteomic analysis study using clinical isolates showed the potential role of iron metabolism in the development of metronidazole resistance [40]. In recent years, Boekhoud et al. discovered the high replication number of pCD-METRO plasmid in *C. difficile* isolates that displayed stable and medium-independent resistance to metronidazole in a large collection of clinical isolates [41]. In our study, we sought to explore whether the presence of iron in the medium could lead to resistance to metronidazole. We found that the addition of Fe^3+^ to growth media at concentrations of 10, 50, and 100 μM resulted in more than eightfold increases in MICs, compared with control media with no additional iron supplementation. The most significant increases occurred at 50 μM in both 24 and 48 h time points in strain 9689. While the folds of increase were significant, the MIC values of MTZ would not be considered resistant as the EUCAST breakpoint is 2 µg/mL, and the CLSI breakpoint is 32 µg/mL. Thus, iron did not lead to MIC values that would be classified as resistant. However, such increases observed in our study may still be clinically relevant. Gonzalez-Luna et al. recently found through classification and regression tree (CART) analysis that MICs higher than 1 µg/mL were significantly associated with clinical failure in the treatment of *C. difficile* [42]. The highly virulent epidemic NAP1/027 *C. difficile* strain R20291 exhibited resistance to metronidazole treatment with MICs >64 µg/mL, independent of media iron supplementation.

During the time of this study, Boekhoud et al. followed up on their medium-independent metronidazole resistance finding, with a discovery of medium-dependent resistance [43]. Their study found that haem presence in media led to an 8- to 25-fold increase in metronidazole MICs, compared with media without haem. Our results also demonstrate that when Fe^3+^ is added to the growth medium, metronidazole MICs increase up to eightfold. Such observation further supports their finding, as haem is an iron-containing porphyrin and would thus lead to their observed increase in MICs with haem supplementation. We further demonstrated that co-treatment with iron chelator DFO effectively obviated the increase in MTZ MICs in the presence of excess iron. Furthermore, our study suggests that the increase in MIC induced by iron was specific to metronidazole at 24 h and 48 h in strain 9689 and to fidaxomicin at 24 h and 48 h in both 9689 and R20291; however, vancomycin MICs were unaffected under the same growth conditions and time points. This observation is similar to what Boekhoud et al. reported, indicating that, despite haem supplementation of growth mediums, no increases in vancomycin MICs were evident, and the effect of haem was only observed with metronidazole treatment [43].

Finally, we found that, in the presence of excess iron, *C. difficile* toxin A production was increasingly measurable by Western Blot, and mRNA levels for *tcdA* and *tcdB* were induced relative to control. The presence of iron increased the measure of toxin A present in media in a dose-dependent manner. However, when normalizing the data to CFU counts from aliquots of the cultures before supernatant preparation, no significant change was observed after Fe^3+^ treatment, compared with the control (Figure 1d). This result suggests that the increased levels of toxin A with Fe^3+^ supplementation were, at least partially, related to the increased CFU of *C. difficile* stimulated by Fe^3+^. The increase in *tcdA* and *tcdB* mRNA levels observed may also be explained by the increased cell density that resulted from the iron supplementation, which could have resulted in quorum sensing, leading to the *tcdA/B* transcriptional increases observed [44].

In recent years, there has been an increased risk of CDI in patients diagnosed with cancer [45]. This association is likely due to the common use of broad-spectrum antimicrobials and extensive chemotherapy, both of which can disrupt the gut microbiome. In addition to the prolonged immunocompromised status in cancer patients, GI toxicities associated with chemotherapy, such as mucositis, may increase the germination of *C. difficile* spores and disease manifestation [46,47]. Given the roles of iron in *C. difficile* as described above, it is interesting to speculate that the excess iron present in patients with cancer after receiving red blood cell transfusions may increase the risk of *C. difficile* infection. The clinical importance of our findings is that iron can affect the susceptibility of *C. difficile* strains to metronidazole and fidaxomicin. Currently, CDI treatment relies on three antibiotics: metronidazole, vancomycin, and fidaxomicin. Vancomycin and fidaxomicin are considered first-line agents in adults, and in pediatrics, vancomycin or metronidazole are recommended treatments [6,48]. Since metronidazole is a therapeutic option for *C. difficile* in pediatric patients, it is crucial to consider the influence of iron overload in the pediatric oncology patient population when determining the course of treatment for CDI. Moreover, as our study showed that in the presence of excess iron, the tested *C. difficile* strains remained susceptible to vancomycin, it provides the rationale for using vancomycin as the first-line treatment of CDI among these patients. These observations on susceptibility, coupled with the observation that Fe^3+^ can directly increase *C. difficile* growth and indirectly lead to more potential toxin production via increased growth, suggests that iron may potentially affect therapeutic outcomes in specific patient populations with iron overload.

Moreover, iron chelation has been shown to reduce iron-induced tissue toxicity and improve patients’ short- and long-term treatment outcomes [49,50]. In our study, co-treatment with iron chelator effectively reduced the toxin levels of *C. difficile* induced by excess iron and resensitized *C. difficile* to MTZ. In recent years, the development of novel iron chelators has attracted more attention as they have shown promising antitumor, antimicrobial, and cardioprotective activities [51,52,53,54,55,56]. Deferoxamine has been used as the standard drug for iron chelation therapy over the past four decades, which requires parenteral administration and presents with significant adverse effects, such as injection site reaction, hypersensitivity reactions, and neurological disturbances. The newer generation of oral iron chelators, such as deferasirox, is more convenient for administration and is suitable for patients with chronic conditions requiring prolonged iron chelation therapy [57]. However, deferasirox may cause acute kidney injury requiring dialysis, severe hepatic toxicity, and gastrointestinal hemorrhage, which requires close patient monitoring. The development of safe and potent iron chelators is greatly warranted, which may also benefit CDI management in iron-overloaded patients with cancer. Further in vivo studies are warranted to determine the effects of iron overload and chelation on CDI using an animal model. The role of iron in persistent and recurrent *C. difficile* infections among patients with cancer also needs to be defined.

## 4. Materials and Methods

### 4.1. Bacterial Strains, Culture Conditions, and Growth Analysis

Two different strains of *C. difficile* were studied: the historic *TcdA* and *TcdB* producing ATCC^®^ 9689™, and PFGE Type NAP1, ribotype 027 strain R20291 (Supplementary Table S1). ATCC^®^ 9689 was purchased from VWR (Wayne, PA, USA). *C. difficile* strain R20291 was a kind gift from Dr. Kevin Garey, University of Houston College of Pharmacy, Houston, TX, USA.

Both *C. difficile* strains were routinely cultured from frozen glycerol stocks (−80 °C) and maintained on brain heart infusion (BHI) media (Hardy Diagnostics, Santa Maria, CA, USA) and agar plates supplemented with 0.5% yeast extract (VWR, Wayne, PA, USA) and 0.1% L-cysteine (Sigma Aldrich, St. Louis, MO, USA) under anaerobic conditions (85% N_2_, 5% H_2_, and 10% CO_2_) in a BACTRON™300 anaerobic chamber (Sheldon Manufacturing Inc., Cornelius, OR, USA). Overnight cultures in BHI broth were started using identical starting inoculation volume and optical density at 600 nm wavelength (OD600) taken using a V-12 Spectrophotometer (VWR).

The growth of *C. difficile* was determined by the colony-forming units (CFUs). For this, 1 mL aliquots of culture were used for the CFU/mL enumeration. All the growth experiments were performed at least three times.

### 4.2. Antibiotics, Iron-Chelators and Iron Supplementation

The stocks of metronidazole (MTZ, metronidazole injection 5 mg/mL, Hospira, Lake Forest, IL, USA) and vancomycin (VAN, vancomycin hydrochloride for injection, Alvogen, Morristown, NJ, USA) were prepared in normal saline. Fidaxomicin (Merck Co.; Kenilworth, NJ, USA) and the iron chelator deferoxamine (Sigma Aldrich Chemical Co.; St. Louis, MO, USA) were reconstituted with sterile water. Exogenous iron was added to growth media as iron (III) chloride (Sigma-Aldrich, St. Louis, MO, USA) in sterile water. All stock solutions were filter-sterilized (0.22 μm) before use.

### 4.3. Susceptibility Testing

*C. difficile* strains were first grown overnight in BHI broth. The susceptibilities to metronidazole (MTZ) or vancomycin (VAN) were then conducted using the broth microdilution method in 96-well round-bottomed plates. BHI broth cultures were diluted to 0.5 McFarland turbidity in normal saline, and a 1:150 dilution was used to inoculate pre-reduced BHI broth. Antibiotic stocks were further diluted in BHI broth with serial twofold dilutions. Negative and positive controls were tested in parallel. Ferric chloride was added at final concentrations of 10, 50, and 100 μM into BHI broth to assess the effect of iron overload on antibiotic susceptibility. In experiments that required an iron chelator, deferoxamine was added to the media at final a concentration of 50 μM. Then, 125 μL of prepared *C. difficile* solution (1:150 dilution) was added to duplicate wells of a 96-well plate containing 125 μL of twofold serially diluted MTZ, VAN, and fidaxomicin. MIC plates were incubated anaerobically at 37 °C, and results were read after 24 h and 48 h incubations using an iMark™ Microplate Reader (Bio-Rad, Hercules, CA, USA). At least three independent experiments with two replicates in each were performed. The MIC was defined as the lowest concentration of antibiotic that prevented visible and measurable (via optical density) bacterial growth after 24 h and 48 h.

### 4.4. Polyacrylamide Gel Electrophoresis (PAGE) and Western Blot Analysis

Iron at various concentrations was added to the bacterial culture to determine the effects of iron on the levels of *C. difficile* toxin production. After 16 h incubation, 10 mL bacterial supernatants were collected, 0.5 mL was used for CFU counting, and the remaining 9.5 mL was centrifuged at 5000× *g* for 10 min and concentrated 10 times using 3K MWCO Amicon^®^ Ultra-15 Centrifugal Filter Unit (Sigma-Aldrich, St. Louis, MO, USA). Equal volumes of clear concentrated supernatants were subjected to SDS–PAGE Western Blot analysis.

Samples in 4 × sample buffer (Bio-Rad, Hercules, CA, USA) were separated by SDS–polyacrylamide (7.5%) gel electrophoresis, followed by transfer onto nitrocellulose membranes (GE Healthcare). After incubation with blocking solution (1 × TBS, 10% non-fat milk, and 0.1% Tween-20) for 2 h at room temperature, the blocked membrane was incubated with primary Anti-*Clostridium difficile* toxin A (PCG4, #ab19953) overnight (4 °C, 1:1000 dilution). Secondary antibodies, anti-mouse-IgG-horseradish peroxidase-linked or anti-rabbit IgG-horseradish peroxidase-linked whole antibodies (Cell Signaling Technology, Danvers, MA, USA), were used at a dilution of 1:5000 in blocking solution. Immunoreactive bands were visualized using the SuperSignal™ West Pico PLUS Chemiluminescent Substrate (Thermo Fisher Scientific, Waltham, MA, USA) and Bio-Rad Imager (Bio-Rad, Hercules, CA, USA).

### 4.5. Chrome Azurol S (CAS) Assay

Antibiotics at different concentrations were subjected to the chrome azurol S (CAS) assay, a colorimetric method that detects siderophore-iron complexes, performed according to Schwyn and Neilands (1987) [33]. Briefly, 500 μL of compound dilutions were added to 500 μL of CAS assay solution (6 mL of 10 mM hexadecyltrimethylammonium bromide (HDTMA), 1.5 mL of 1 mM FeCl_3_, 7.5 mL of 2 mM CAS, 4.307 g of piperazine, and 6.25 mL of 12 M HCl diluted to 100 mL in deionized water; final pH of 5.6) and incubated for 1 h at room temperature with gentle mixing. Absorbance spectra for CAS solution–supernatant mixtures were then measured at 630 nm using a V-12 Spectrophotometer (VWR, Wayne, PA, USA). Iron chelator DFO and ferrichrome, a siderophore produced by *Ustilago sphaerogena*, were positive controls.

### 4.6. Isolation of RNA and qRT-PCR Assay

Total RNA was isolated from collected bacterial pellets using the using Quick-RNA™ Miniprep kit (Zymo Research, Irvine, CA, USA) according to the manufacturer’s instructions and quantified using Nanodrop. Changes in *tcdA* and *tcdB* gene expressions were assessed by quantitative real-time PCR using methods previously described with *C. difficile* 16S rRNA as the internal control. The primers were *tcdA*-forward, CAACACCTTAACCCAGCCATA; *tcdA*-reverse, AGAGTTTTCTGCGGTAGCTGA; *tcdB*-forward, ATCTGGAGAATGGAAGGTGGT; *tcdB*-reverse, TGATGGTGCTGAAAAGAAGTG; 16S rRNA-forward, AGCGGTGAAATGCGTAGATAT; 16S rRNA-reverse, CAG CGTCAGTTACAGTCCAGA [28]. First-strand cDNA was prepared from 500 ng RNA using the iScriptTM cDNA Synthesis Kit (BioRad; Hercules, CA, USA). cDNA synthesis was carried out in C1000 Touch™ Thermal Cycler (BioRad) with priming at 25 °C for 5 min, reverse transcription at 50 °C for 10 min, followed by iScript reverse transcriptase inactivation at 95 °C for 5 min. Quantitative PCR was performed using SsoAdvanced Universal SYBR Green Supermix (BioRad, Hercules, CA, USA) in CFX96 Touch™ Real-Time PCR Detection System (BioRad, Hercules, CA, USA). PCR amplification of cDNA (1 µg) was performed under the following conditions: 95 °C for 10 min; 40 cycles at 95 °C for 15 s, followed by 55 °C for 60 s. Relative gene expression was determined using the 2^−∆∆Ct^ method. The sample’s mean value of Ct of 16S rRNA (internal control gene) was subtracted from the sample mean Ct of the *tcdA* and *tcdB* genes (∆Ct), respectively. ∆Ct of the no treatment control was subtracted from the mean ∆Ct of each experimental sample (∆∆Ct). This 2^−∆∆Ct^ method yields the fold of change in gene expression of the gene of interest normalized to the expression of the 16S rRNA internal control and relative to the no treatment control.

### 4.7. Statistical Analysis

Data are presented as numbers and percentages for categorical variables and as means ± standard deviation for continuous variables. Statistical analyses were performed with *t*-tests for independent variances. Two-sided *p*-values of <0.05 were considered significant.

## 5. Conclusions

Our study demonstrates that the growth of *C. difficile* and toxin measured was significantly increased by excess iron. The presence of excess iron also contributed to the increasing MICs of metronidazole and fidaxomicin. The increase in MICs for metronidazole was significant for the classic 9689 strain and for fidaxomicin MICS in the hypervirulent R20291 strain. Using an iron chelator led to a reversal of the observed effects and thus may serve as a unique strategy for CDI treatment in patients in whom iron overload is suspected or present.

## Data Availability

The data presented in this study are available in the manuscript and the supplemental materials. Additional information may be requested from the corresponding author.

## Supplementary Materials

The following supporting information can be downloaded at: https://www.mdpi.com/article/10.3390/antibiotics11050537/s1, Figure S1: Effect of Fe^3+^ on colony formation units in *C. difficile* R20291 strain, Table S1: Summary of characteristics of *C. difficile* strains used in the study, Table S2: Effects of Fe^3+^ on the MICs of metronidazole in different *C. difficile* strains, Table S3: Effects of Fe^3+^ on the MICs of vancomycin in different *C. difficile* strains, Table S4: Effects of Fe^3+^ on the MICs of fidaxomicin in different *C. difficile* strains.
