# Supplementary material for: Iron Effects on Clostridioides difficile Toxin Production and Antimicrobial Susceptibilities
Source: Antibiotics (Basel). 2022 Apr 19;11(5):537. doi: 10.3390/antibiotics11050537 (PMC9137654; doi:10.3390/antibiotics11050537)
Supplement: Supplementary file 1 [file antibiotics-11-00537-s001.zip › antibiotics-1660961-supplementary.pdf]

Supplemental Tables and Figures

Figure S1. Effect of Fe^{3+} on colony formation units in *C. difficile* R20291 strain

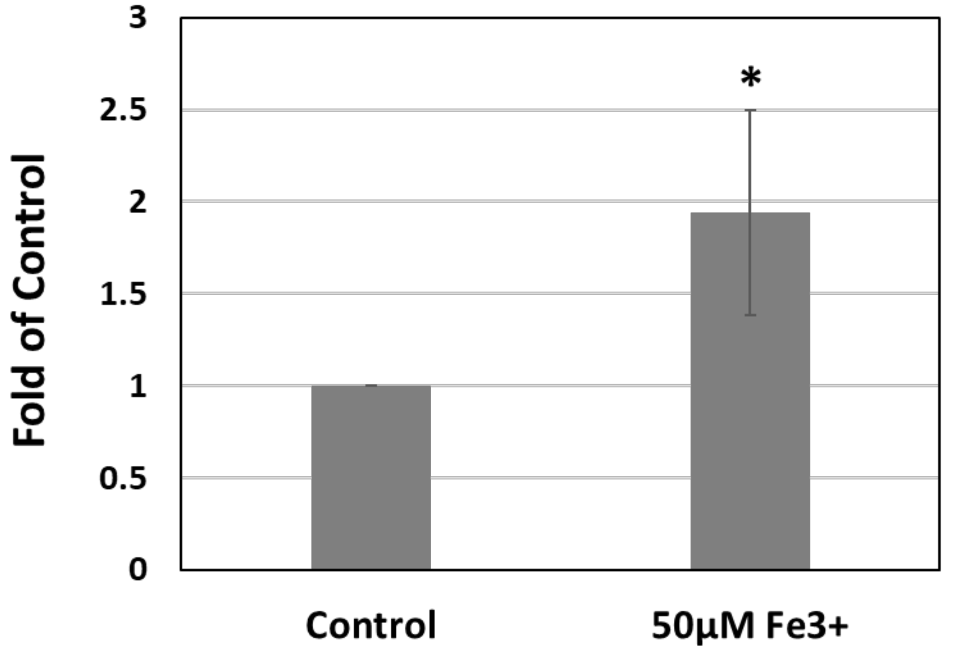

Table S1. Summary of characteristics of *C. difficile* strains used in the study

	**ATCC® 9689**	***Peptoclostridium difficile* (strain R20291)**
**Type strain**	Yes	No
**Toxinotype**	tcdA+/tcdB+	tcdA+/tcdB+
**Ribotype**	1	27
**Binary Toxin gene cdtB by PCR**	Negative	Positive
**Toxigenic**	Yes	Yes, hypervirulent, PFGE type NAP1

Table S2. Effects of Fe^{3+} on the MICs of metronidazole in different *C. difficile* strains

	Average MIC metronidazole (ug/ml)							
	Control		10 µM Fe3+		50 µM Fe3+		100 µM Fe3+	
C. difficile strain	24hr	48hr	24hr	48hr	24hr	48hr	24hr	48hr
ATCC® 9689™	0.16	0.17	0.38	0.71	0.77	1.38	0.44	1.13
R20291	>8	>8	>8	>8	>8	>8	>8	>8

Table S3. Effects of Fe^{3+} on the MICs of vancomycin in different *C. difficile* strains

	Average MIC Vancomycin (ug/ml)							
	Control		10 μM Fe3+		50 μM Fe3+		100 μM Fe3+	
C. difficile strain	24hr	48hr	24hr	48hr	24hr	48hr	24hr	48hr
ATCC® 9689™	0.75	1.00	0.75	1.00	0.75	1.00	0.63	0.88
R20291	0.50	1.00	0.50	1.00	0.50	1.00	0.50	1.00

Table S4. Effects of Fe^{3+} on the MICs of fidaxomicin in different *C. difficile* strains

	Average MIC Fidaxomicin (ug/ml)							
	Control		10 µM Fe3+		50 µM Fe3+		100 µM Fe3+	
C. difficile strain	24hr	48hr	24hr	48hr	24hr	48hr	24hr	48hr
ATCC® 9689™	0.03	0.54	0.03	0.42	0.21	1.71	0.03	0.38
R20291	1.00	8.00	1.00	13.30	1.00	16.00	1.00	8.00
